# Optimizing Nutrition Education in a Dedicated Inflammatory Bowel Disease Clinic

**DOI:** 10.1093/crocol/otaf045

**Published:** 2025-07-10

**Authors:** Thomas W Fredrick, June Tome, Camille A Kezer, Krista R Kerlinske, Lindsey E Sefried, Sunanda V Kane

**Affiliations:** Division of Gastroenterology and Hepatology, Mayo Clinic, Rochester, MN, United States; Division of Gastroenterology and Hepatology, Mayo Clinic, Rochester, MN, United States; Division of Gastroenterology and Hepatology, Mayo Clinic, Rochester, MN, United States; Division of Gastroenterology and Hepatology, Mayo Clinic, Rochester, MN, United States; Division of Gastroenterology and Hepatology, Mayo Clinic, Rochester, MN, United States; Division of Gastroenterology and Hepatology, Mayo Clinic, Rochester, MN, United States

**Keywords:** inflammatory bowel diseases, nutritional status, diet, food, and nutrition, quality improvement

## Abstract

**Background:**

Patients with inflammatory bowel disease (IBD) frequently ask their providers for nutritional or dietary recommendations; however, providers are limited in both time and knowledge to adequately address their questions. In this single-center study, we sought to improve provider experiences with nutrition counseling for patients seen in a dedicated IBD clinic.

**Methods:**

To understand the current state, providers, including gastroenterology fellows, attendings, and advanced practice providers were surveyed regarding their experiences with nutritional recommendations in a dedicated IBD clinic. Following the pre-intervention survey, we worked with registered dieticians on how to address key concerns and developed an informational handout based upon current guidelines. After displaying handouts in clinical workspaces for 5 weeks, providers were surveyed again to evaluate their response.

**Results:**

All 22 respondents (100%) in the pre-intervention survey either agreed or strongly agreed that IBD patients have unique nutritional requirements. A majority (72%) strongly agreed that the clinic would benefit from more access to dieticians. Additionally, 41% of providers either strongly disagreed or disagreed that they had enough time to address nutritional concerns. Post-intervention, 57% of respondents (8/14) reported that they found the handouts helpful. A significant number of providers reported improvement in their comfort level discussing nutrition and dietary recommendations with IBD patients, with tmean Likert score increasing from 3.5 to 4.1 (*P* = .01).

**Conclusion:**

In this quality improvement study, we identified key issues preventing providers from addressing patient desire for nutritional counseling and developed a novel awareness campaign that significantly improved provider confidence in discussing nutritional recommendations with their IBD patients.

## Article Summary

This study identified key issues impacting patient experiences with nutritional and dietary counseling at a dedicated inflammatory bowel diseases (IBD) center. By implementing an informational campaign, we significantly improved provider confidence in discussing nutrition recommendations with patients with IBD.

## Introduction

Malnutrition in inflammatory bowel disease (IBD) has a significant impact on patient quality of life and has been associated with higher health-care utilization as well as longer length of hospitalization.^1^^,^^2^ Even in the absence of malnutrition, many patients with IBD seek dietary and nutritional advice from their IBD provider. Studies have demonstrated that a substantial proportion of patients, up to 71%, believe that diet has an important influence on their IBD and could affect their symptoms in addition to risk of disease relapse.^3^^,^^4^ In contrast, over half of patients have reported in various questionnaire studies that they felt their IBD provider disregarded the importance of diet and nutrition at clinic visits.^3^ In addition, the advice given to patients is frequently heterogeneous and inconsistent regarding topics such as probiotics, role of low residue diets, role of soluble and insoluble fiber, and specific elimination diets.

There may be several reasons for this perceived difference between patients and providers regarding the role of nutrition in IBD, including appointment time constraints, lack of formal training for providers regarding specific nutritional and dietary recommendations for ulcerative colitis and Crohn’s disease patients, and potential deferral of questions to registered dietitians. Given that the role of diet for patients with IBD can be a source of uncertainty for both providers and patients, the American Gastroenterological Association (AGA) recently released clinical practice guidelines on diet and nutritional therapies for IBD.^5^ These guidelines provide tailored approaches depending on IBD disease activity, severity, and character.

In our IBD Center, we observed that patients with IBD frequently ask their providers for nutritional or dietary recommendations. We hypothesized that providers may have limited time and knowledge to adequately address their questions. In this single-center quality improvement study in a dedicated IBD clinic we sought to both evaluate and improve provider experiences with nutritional counseling.

## Methods

**Figure 1.**
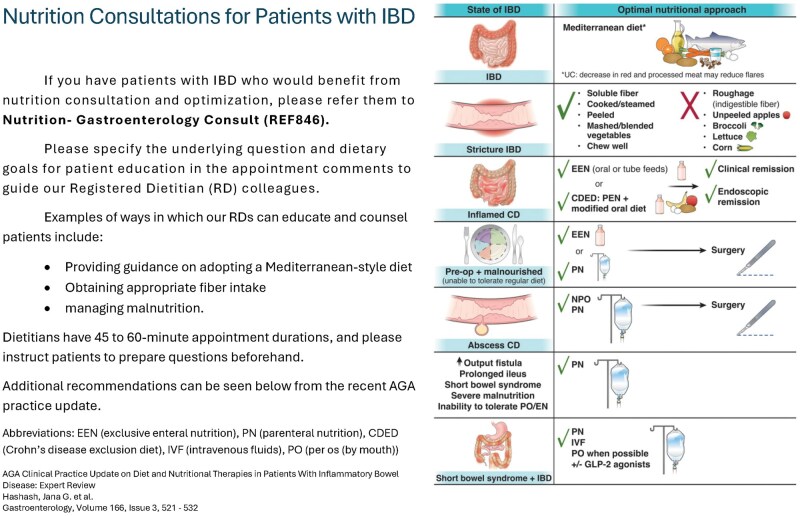
Informational nutritional handout.

To understand the current state of nutritional care in a dedicated IBD clinic, providers including gastroenterology fellows, attendings, and advanced practice providers were surveyed about their views regarding nutritional management of patients with IBD (Table 1). Responses were graded on either yes/no or with a 5-point Likert scale. Following the pre-intervention survey, we strategized with registered dietitians about how to address key concerns expressed by providers. From this discussion, we created an informational handout (Figure 1) based upon current guidelines for nutritional management in IBD.^5^^,^^6^ Handouts were provided to all practitioners and displayed in clinical workspaces for both providers and patients to view.

**Table 1.** Provider responses regarding nutritional management of patients with IBD before and after study intervention.

**Question**	Pre-­intervention (*n* = 22)	Post-intervention (*n* = 14)
IBD patients have unique nutritional requirements	4.4 (0.5)	**Not surveyed**
I have adequate time to discuss nutrition and dietary recommendations with my IBD patients	2.9 (0.98)	**Not surveyed**
My patients with IBD have expressed a desire to meet with a nutrition expert	4.1 (0.87)	**Not surveyed**
I feel comfortable discussing dietary recommendations for my patients with IBD	3.5 (0.89)	4.1(0.26), *P* = 0.01
I have found the outpatient dietitian visits are helpful	3.3 (1.17)	3.6 (0.61), *P* = 0.24
My patients have reported meeting with a dietitian was helpful	3.3 (1.05)	3.4 (0.49), *P* = 0.68
The IBD clinic would benefit from dietitians specifically trained in patients with IBD	4.7 (0.55)	4.7 (0.59), *P* = 0.87
I recall seeing the informational nutrition flyers and/or emails	**Not surveyed**	*N* = 10 (71%)
I found the informational nutrition flyers/emails helpful	**Not surveyed**	3.7 (1.22)

Responses listed as mean (SD) Likert score (1 = strongly disagree, 5 = strongly agree) unless otherwise stated.

Abbreviation: IBD, inflammatory bowel disease.

After the study period of 5 weeks, providers were surveyed again to evaluate their experience with nutritional management of patients with IBD post-intervention. Changes in survey responses were analyzed using Student’s *t*-test. *P* < .05 were considered statistically significant. Assessment of IBD clinic visits associated with nutrition referrals was performed over a defined 3-month period both after our intervention and compared to the same 3-month period 1 year previously. This study was deemed exempt by the Mayo Clinic institutional review board.

## Results

A total of 22 providers completed the pre-intervention survey. 100% of respondents in the pre-intervention survey either agreed (*n* = 12) or strongly agreed (*n* = 10) that patients with IBD have unique nutritional requirements, and 72% (16/22) strongly agreed that the clinic would benefit from more access to registered dietitians. 41% of providers either strongly disagreed (1/22) or disagreed (8/22) that they had enough time to address nutritional concerns, and 50% either were neutral (8/22) or disagreed (3/22) that they felt comfortable discussing nutritional recommendations with patients with IBD.

This feedback was provided to registered dietitians to discuss optimal nutritional management of patients with IBD. Based on this discussion, it was determined that education of providers of the specific services the dietitians recommend for patients with IBD would be most helpful for addressing the nutritional concerns of patients with IBD. This led to generation of an informational handout (Figure 1) which was used to help educate providers in the IBD clinic and set patient and provider expectations prior to a dietitian visit.

Fourteen providers completed the post-intervention survey, with 70% (10/14) of respondents recalling viewing the handouts and 57% (8/14) reporting that they found the handouts helpful. There was a significant increase in provider comfort in discussing nutrition and dietary recommendations with IBD patients, reflected by a mean Likert score increasing from 3.5 to 4.1 (Table 1, *P* = .01).

One year prior to the intervention, 4.90% and 2.80% of all gastroenterology clinic visits and IBD-specific visits, respectively, resulted in a dietitian referral. After the intervention, a total of 3.90% and 2.60% of all gastroenterology visits and IBD-specific visits, respectively, included a dietitian referral.

## Discussion

In this quality improvement study, we identified key issues impacting the ability of providers to address patient desire for nutritional counseling in IBD. By collaborating with registered dietitians, we developed informational handouts to guide providers in addressing key nutritional issues in this patient population. Providers overwhelmingly agreed that patients with IBD had unique nutritional requirements, a desire to meet with a nutrition specialist, and a belief that the clinic would benefit from dietitians trained specifically in IBD management. Use of a nutritional handout for providers significantly improved provider confidence in discussing nutritional recommendations with patients with IBD.

While our gastroenterology department experienced a decrease in dietitian referrals practice-wide from the year before this intervention, this decrease was attenuated in our IBD clinic after this intervention. Further investigations are needed to evaluate factors that might be responsible for this practice-wide drop in dietitian referrals.

Strengths of the project include the diverse perspectives of providers surveyed, including gastroenterology trainees and advanced practice providers, as well as the feasibility of this intervention that can be easily adopted at other institutions. Limitations are the small cohort size and that the project was conducted at a single academic center, which may limit the generalizability of the results.

In summary, gastroenterology providers benefit from education on how to provide evidence-based nutritional recommendations to patients with IBD. The AGA guideline from 2024 provides clear, synthesized dietary recommendations for patients with IBD that providers found helpful. Our study focused on the provider experience in order to ensure nutritional consults were optimized, and evaluation of the patient experience as well as outcomes resultant of nutritional counseling in future studies will be essential to make sure all patients with IBD have their needs addressed.

## Patient Synopsis

This study looked at how providers can best address nutrition and diet recommendations for patients with IBD. We designed an informational handout for providers that improved their confidence in discussing nutritional recommendations.

## Supplementary Material

otaf045_Supplementary_Data

## Data Availability

Data available in supplementary material.
